# Assessment of barriers and facilitators in the implementation of appropriate use criteria for elective percutaneous coronary interventions: a qualitative study

**DOI:** 10.1186/s12872-018-0901-6

**Published:** 2018-08-13

**Authors:** Anne Lambert-Kerzner, Charles Maynard, Marina McCreight, Amy Ladebue, Katherine M. Williams, Kelty B. Fehling, Steven M. Bradley

**Affiliations:** 1 VA Eastern Colorado Health Care System, Department of Veterans Affairs Medical Center, Denver, CO USA; 2 School of Public Health, University of Colorado, Aurora, CO USA; 3 Foundation for Health Care Quality Clinical Outcomes Assessment Program, Seattle, WA USA; 4 Minneapolis Heart Institute, Minneapolis, MN USA

**Keywords:** Appropriate use criteria, Qualitative, Ischemic heart disease, Percutaneous coronary intervention

## Abstract

**Background:**

The use of inappropriate elective Percutaneous Coronary Intervention (PCI) has decreased over time, but hospital-level variation in the use of inappropriate PCI persists. Understanding the barriers and facilitators to the implementation of Appropriate Use Criteria (AUC) guidelines may inform efforts to improve elective PCI appropriateness.

**Methods:**

All hospitals performing PCI in Washington State were categorized by their use of inappropriate elective PCI in 2010 to 2013. Semi-structured, qualitative telephone interviews were then conducted with 17 individual interviews at 13 sites in Washington State to identify barriers and facilitators to the implementation of the AUC guidelines. An inductive and deductive, team-based analytical approach, drawing primarily on Matrix analysis was performed to identify factors affecting implementation of the AUC.

**Results:**

Specific facilitators were identified that supported successful implementation of the AUC. These included collaborative catheterization laboratory environments that allow all staff to participate with questions and opinions; ongoing AUC education with catheterization laboratory teams and referring providers; internal AUC peer review processes; interventional cardiologist be directly involved with the pre-procedural review process; checklist-based algorithms for pre-procedural documentation; systems redesign to include insurance companies; and AUC educational information with patients. Barriers to implementation of the AUC included external pressures, such as competition for patients, and the lack of shared medical records with sites that referred patients for coronary angiography.

**Conclusions:**

The identified facilitators enabled sites to successfully implement the AUC. Catheterization laboratories struggling to successfully implement the AUC may consider utilizing these strategies to improve their processes to improve patient selection for elective PCI.

## Background

The Appropriate Use Criteria (AUC) for Coronary Revascularization reflects a collaborative effort by professional societies to evaluate and support optimal patient selection for percutaneous coronary intervention (PCI) [1, 2]. Prior application of these criteria suggest as many as one in six elective coronary procedures are performed for inappropriate indications, defined as the procedural risk outweighing anticipated patient benefit [3–5]. Since release of the AUC in 2009, the numbers of inappropriate PCIs have decreased, consistent with improved patient selection for the procedure [3, 4, 6]. However, these improvements have not been uniform; some hospitals have achieved large reductions in the inappropriate use of elective PCI, while for other hospitals, rates of inappropriate PCI remain unchanged [3, 4, 6].

Understanding the interplay among clinical processes, clinical providers’ behaviors, and the organizational context in which they occur has been shown to inform and improve other healthcare processes [7]. Similarly, identifying and understanding factors that contribute to the successful adoption and implementation of the AUC at individual hospitals may inform strategies associated with improvements in patient selection for elective PCI. These factors may help suboptimal performing hospitals achieve improvements seen at hospitals with reductions in the inappropriate use of elective PCI.

Accordingly, we performed an analysis within a statewide quality improvement program for coronary revascularization to identify factors associated with patient selection and inappropriate use of elective PCI at the hospital level. Using data from the Washington State Clinical Outcomes Assessment Program (COAP) [8, 9] we previously described temporal trends in the number of PCI procedures performed, their appropriateness as determined by application of the AUC, and identified high, medium, and low-performing hospitals defined by a low overall proportion or a declining temporal proportion of elective PCI classified as inappropriate [11]. The objective of the present investigation was to conduct a qualitative evaluation to identify and assess healthcare system strategies to support the implementation of the guidelines and important contextual factors to minimize inappropriate patient selection for elective PCI.

## Methods

### Study setting

COAP is a quality-improvement initiative of the Foundation for Health Care Quality, a non-profit 501(c)3 corporation, designed to improve quality of care for patients receiving cardiac interventions in the state of Washington [8, 9]. Using data from COAP, a quantitative analysis provided information on rates of inappropriate elective PCI and hospital categories of procedural appropriateness [10]. Hospital tertiles of appropriateness performance were based on average rates of inappropriate elective PCI and reduction in rates of inappropriate elective PCI over time. High performing hospitals were either in the tertile of hospitals with the smallest proportion of inappropriateness or highest tertile of reduction in inappropriate elective PCI over time. Low performing sites were those in hospital tertile with the largest proportion of inappropriate elective PCI or smallest reduction in inappropriate elective PCI over time, and medium sites were in the middle of the high and low sites.

Informed by this prior quantitative analysis, we conducted a qualitative inquiry of individual sites that allowed us to gain a comprehensive understanding and identification of factors that contributed to successful adoption and implementation of the AUC. In addition, identification of barriers that impeded successful implementation of the AUC at specific sites provided insights for improvement. Qualitative inquiry allowed us to explore further why and how these factors were implemented better than quantitative surveys. The Colorado Multiple Institutional Review Board approved this study and a waiver of informed consent was granted.

### Data collection

A purposive sample [12] of 16 sites that represented each level of performance was identified by the quantitative team and COAP. We requested a roughly equal number of sites in each of the tertiles of appropriateness performance. Contact information for the identified sites’ cardiac catheterization laboratories (cath lab) was obtained from COAP by the study principal investigator (SB). We pursued contacts in quality improvement and cardiologist roles. Assertive attempts were made to contact all sites included multiple emails sent by the study PI with follow-up phone calls and additional emails sent by the project coordinator.

**Fig. 1:**
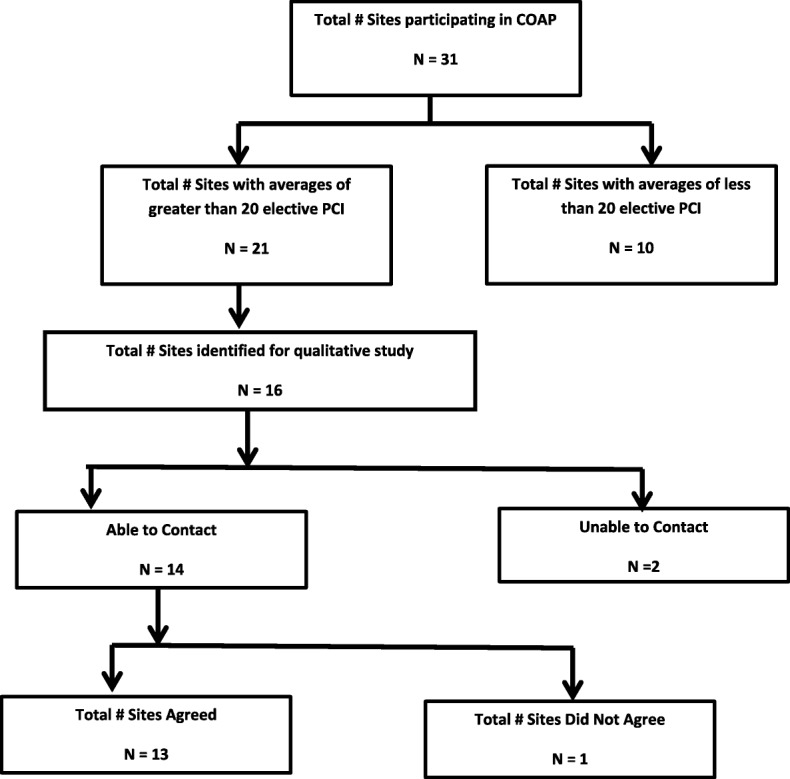
Flow chart of site qualitative interviews

Those sites who agreed to be interviewed identified an initial contact. The project coordinator contacted each person to schedule their telephone interviews. This process continued and expanded utilizing an assertive snowball technique throughout the interviews to identify others within the same site. Of the 16 sites identified, 13 agreed to participate in the qualitative inquiry. We identified 5 sites as high performing sites, 4 as low performing sites, and 4 fell between the high and low criteria. Of the three sites not interviewed 2 were high performing sites and 1 was a medium to high performing site. A flow chart of hospital participation is provided in Fig. 1. Hospital characteristics are provided in Table 1 including the 3 sites not interviewed. Of the 33 individual contacts provided, 17 responded and scheduled an interview whereas the remaining contacts never responded the interview requests or declined to be interviewed. At the 13 sites interviewed, we conducted one-to-one telephone interviews, which lasted about 40 to 60 min with the 17 participants, including 6 in administrative roles, 10 interventional cardiologists, and one nurse manager. Participant demographics are provided in Table 2.

**Table 1:** Hospital characteristics

Site	PCI volume	Bed size	Location	On site surgery
High performance Hospital				
# 1	831	501	Urban	Yes
# 2	467	347	Urban	Yes
# 3	230	213	Urban	Yes
# 4	473	291	Urban	Yes
# 5	173	226	Suburban	No
Median or %	467	291	80% urban	80% on site surgery
Medium performance Hospital				
# 6	1291	644	Urban	Yes
# 7	286	156	Suburban	No
# 8	533	312	Urban	Yes
# 9	616	318	Suburban	No
Median or %	574.5	315	50% urban	50% on site surgery
Low performance Hospital				
#10	95	110	Suburban	No
# 11	739	450	Urban	Yes
# 12	844	343	Suburban	Yes
# 13	502	254	Suburban	Yes
Median or %	620.5	298.5	75% suburban	75% on site surgery
Sites Not Contacted				
#14	509	262	Suburban	Yes
#15	292	137	Suburban	No
#16	524	253	Suburban	Yes

*PCI* Percutaneous Coronary Intervention

**Table 2:** Participant demographics

Roles	Site	Gender	Educational Attainment
Administrator and Provider (*N* = 1)	1	Male (*N* = 1)	MD (*N* = 1)
Administrator (*N* = 1)	2	Male (*N* = 1)	MD (*N* = 1)
Provider (*N* = 1) Provider (*N* = 1)	3	Female (*N* = 1) Female (*N* = 1)	RN (*N* = 1) MD (*N* = 1)
Provider (*N* = 1)	4	Male (*N* = 1)	MD (*N* = 1)
Provider (*N* = 1) Administrator (*N* = 1)	5	Male (*N* = 2)	PharmD (*N* = 1) MD (*N* = 1)
Provider (*N* = 1)	6	Male (*N* = 1)	MD (*N* = 1)
Provider (*N* = 1)	7	Male (*N* = 1)	MD (*N* = 1)
Provider (*N* = 1)	8	Male (*N* = 1)	MD (*N* = 1)
Administrator and Provider (*N* = 1) Administrator (*N* = 1)	9	Male (*N* = 1) Female (*N* = 1)	MD (*N* = 1) RN (*N* = 1)
Administrator (*N* = 1) Provider (*N* = 1)	10	Male (*N* = 2)	RT (*N* = 1) MD (*N* = 1)
Provider (*N* = 1)	11	Male (*N* = 1)	MD (*N* = 1)
Administrator (*N* = 1)	12	Male (*N* = 1)	Unknown
Administrator (*N* = 1)	13	Female (*N* = 1)	RN (*N* = 1)

Semi-structured interviews were recorded and transcribed verbatim. Sample interview guide question are shown in Table 3. The interviews were conducted by the 5 Masters’ or PhD educated members of the trained qualitative team (ALK, MM, KM, DBF). The interviewers were blinded to the level of appropriateness to reduce interviewing bias (i.e. high, medium, and low performing hospitals).

**Table 3:** Qualitative interview guide domains with example items

Domains	Primary and Secondary Items
Role in the facility related to elective PCI:	
General role	What is your title and function at your facility?
Specific clinical role	Are you presently practicing in the cath lab?
Description of the process/steps from referral through scheduling of a patient for an elective coronary procedure:	
Referral process	Please describe the process of how patients are referred for a coronary procedure?
	What do you think is important to do prior to the cath lab to help ensure the necessity of the coronary procedure?
	How do you obtain this data/medical records?
	What is your opinion of the current process for patient referral for elective coronary angiography and PCI?
	Are there differences in how patients are referred from providers outside of the center?
	Are referrals for coronary procedures reviewed prior to scheduling?
	Does the cath lab contact the patient after the referral but before the procedure has been scheduled?
Challenges in the referral process	Can you think of practical issues or pressures that make it challenging to ensure a patient is ready for a coronary procedure?
	Are there pressures to accept referrals for coronary procedures? • Probe to describe details
History of referral process	Please tell us how these referral processes were developed?
	If a change in the referral process has occurred in the past few years: can you tell me what the impact has been?
Description of the processes involved after a patient has been scheduled for an elective coronary angiogram or PCI:	
Challenges	Can a scheduled coronary procedure be cancelled prior to a patients’ arrival at the facility? • Probe to describe details
General questions about cath lab:	
Contextual factors	Please describe the working environment, the culture, in your cath lab?
	Please tell us about the involvement hospital administrators, such as quality officers and financial officers, have in patient referrals for elective coronary procedures?

### Data analysis

An inductive and deductive, team-based analytical approach, drawing primarily on Matrix analysis [13, 14] was performed. A Matrix analysis is a tabular format that collects and arranges data for easy viewing in 1 place, permits detailed analysis, and sets the stage for later cross-case analysis with other comparable sites [13, 14]. The validity and accuracy/reliability of codes in the initial codebook were established by each team member who analyzed 2 initial transcripts and eventually achieved consensus [12–15]. Emergent codes were then added throughout the analysis. Remaining interviews were divided among the team to complete the Matrix analysis. Due to the large amount of data, the team continued the analysis utilizing Atlas.ti (Scientific Software Development GmbH) software. Upon thematic saturation (not hearing any new information), which resulted in the creation of domains the team concluded the analysis with multiple meetings reassessing the identified domains that were placed into a final matrix for comparative analysis. Consistency of coding/interpretation was regularly checked with discrepancies resolved through team consensus. Analyses continued with emergent themes, categories, and conclusions [12–15]. Further rigor included: an inter-coder agreement based on coding similar passages with consistent codes with an agreement of 80%; and the creation of an audit trail to document analytical process [12–15].

## Results

This study investigated end-user opinions and perspectives on factors affecting the use of the AUC in individual hospitals. To accentuate the healthcare system strategies and important contextual factors that minimize inappropriate patient selection for elective PCI, we focused the results on the differences between the high and low performing sites. Medium level sites descriptions are included to demonstrate the transition process and the factors that support the movement between high and low performing sites.

As shown in Table 1, the five high performing sites were located in urban settings (80%), had bed sizes ranging from 213 to 501 (median 291), and annual PCI volumes ranging from 173 to 831 (median 467). The four medium sites were equally distributed between urban and suburban settings, with beds sizes ranging from 156 to 644 (median 315), and PCI volumes ranging between 286 and 1291 (median 574.5). The four low sites were predominately in the suburbs (75%), with bed sizes ranging from 110 to 450 (median 298.5) and PCI volumes ranging from 95 to 844 (median 620.5).

Six overarching themes of factors that identify barriers and facilitators in hospitals’ implementation of elective PCI emerged: 1) Relationships and Competition; 2) Appropriate Referrals; 3) Responsibility for Pre-Procedural Process; 4) Documentation of Referral Review, 5) Pressures (External/Internal) and Education, and 6) Peer Review Processes. Table 4 describes the facilitators and barriers.

**Table 4:** Barriers and Facilitators to the implementation of AUC

Theme	High Sites	Medium Sites	Low Sites
Facilitators to the Implementation of AUC			
Relationships and Competition	Described strong collaborative environments	Described good environments	Described “friendly relationships”
Responsibility for Pre-Procedural Processes	Interventional cardiologists were often directly involved in the pre-procedural review of each case	Variety of processes	Leaned towards the referring cardiologist having the responsibility for the PCI appropriateness
Documentation of Referral Reviews	Established ways to ensure documentation, usually with the interventional cardiologists taking the responsibility for such documentation.	1/2 had formal documentation processes	Obligation of the referring physician to work the patient up appropriately
Pressures (External/Internal) and Education	Education was used to improve appropriateness of PCIs within multiple organizations. Educating patients -Educating the internal staff of the AUC		
Peer Review Process	Developed their own peer review processes to ensure appropriateness, team communication, and proper documentation	None of the medium performing sites described any type of peer-review processes.	None of the low sites mentioned peer review processes
Barriers to the Implementation of AUC			
Appropriate Referrals	Most referrals from inside organization	Variety of referrals	More referrals from providers outside their organization compared to medium and high performing sites
Documentation of Referral Reviews	See above for facilitator	See above for facilitator	Lack of staff or other staff responsible extracting data from medical record, and separate medical records systems
Pressures (External/Internal) and Education	Patient pressure -patient satisfaction was very important Difficulty to get outside referring physicians up to speed with AUC		

### Relationships and competition

Collaborative environments were described by the high performing sites. Participants shared that all staff were encouraged to partake in the cath lab process management discussions and were encouraged to ask questions, provide opinion with a focus on patients’ needs and their having positive experiences. The work environments were described as a good collegial atmosphere with positive, supporting, and knowledgeable staff. One site described group meetings every 2 weeks for cath lab conferences and quarterly for mortality reviews where all staff was encouraged to participate. This allowed for more checks and balances among interventional cardiologists and staff.

*“It is a very collaborative culture where the cath lab tech, nurses, and anybody else working in the cath lab have the ability to ask questions and stop the procedure at any time if they have any concerns and that is true from before bringing a patient to the cath lab, during the cath lab procedure, and after the procedure…” (Site #1).*

Some participants stressed it was important to maintain collaborative relationships with referring providers, as one site indicated that AUC improved communication with referring primary care providers.

Medium sites described good working environments, with one site describing tension in failing to get physicians input on things. Low performing sites described the importance of *“friendly relationships”,* and one site expressed there was an opportunity to improve relationships between referral cardiologists and the interventional cardiologists, to provide better communication and education regarding appropriate referrals.

Low performing sites had more competition for patients compared to most high performing sites whose patients were referred from cardiologists inside their organization.

### Appropriate referrals

Obtaining appropriate referrals for patients needing elective PCIs emerged as an important factor for both high and low performing sites. Low performing sites seemed to have more referrals from providers outside their organization compared to medium and high performing sites, which presented greater barriers to obtaining the needed information, especially due to multiple electronic health record systems.

*“Well, we have a 5 county service area. And there are clinics in different communities in those 5 counties, and we have 2 groups of cardiologists in town.” (Site # 12).*

Obtaining the pre-procedural information needed to determine if a patient was a suitable candidate was identified as a barrier for all sites. High performing sites identified that appropriate screening must be done from the beginning of the process and is “*key to selecting the right cohort of patients*”.

### Responsibility for pre-procedural processes

At high performing sites, interventional cardiologists were often directly involved in the pre-procedural review of each case. At multiple sites the interventional cardiologist reviewed the case prior to seeing the patient in the cath lab and met with patients. The interventional cardiologist made sure the patients were adequately evaluated and had undergone proper testing before the procedure. A high-performing site stated the referring cardiologists were aware of AUC guidelines and this process was supported by the use of common electronic medical records (EMR). Yet, another high performing site believed it was the responsibility of the referring cardiologist, with most referrals originating from their own group, which had an extensive common EMR.

Two of the four medium sites stated that their referral process was in transition; *“evolving”* and another thought it could improve with less *“middle men”* involved. The others indicated that interventionalists were personally involved with the process.

The low performing sites had more outside referrals and were more sensitive to not offending the referring cardiologists. They also leaned towards the referring cardiologist having the responsibility for determining PCI appropriateness. One site indicated there were no specific criteria for a review prior to the procedure, no standard processes were in place, and outside referrals varied greatly.

### Documentation of referral reviews

Documentation was identified by most sites as an important factor for assessing appropriateness. As an interventional cardiologist said *“Playing the game is just making sure that all the documentation is there*.” (Site # 4) High sites have established ways to ensure documentation, usually with the interventional cardiologists taking the responsibility for such documentation.

“*Takes a lot of manpower to report the data accurately for COAP. Need to clearly document the risk and best person to fill out this documentation is a physician who understands risk.”* (Site # 3).

High performing sites described specific facilitators to comply with appropriate documentation. One site devised a pre-PCI scoring methodology for determining categorization by the AUC. Another site utilized the Society for Cardiovascular Angiography and Interventions (SCAI) website calculator and still another site used a more granular documentation of stress test results, rather than just normal vs abnormal. EMRs facilitated obtaining and documenting the needed information for appropriateness assessment.

Two of the four medium sites described having more formal processes to document the referral process and two did not have any formal documentation processes in place.

Although some low sites indicated they had similar procedures as the high sites, they also explained that performing all of the recommended preliminary tests may not be the best practice. As a provider at a low performing site explained,

“*And it used to be that that was a very clear thing that everyone kind of knew that that you would do, okay well this guy, you know, walks like a duck, quacks like a duck it’s a duck. And nowadays you get a stress test but I really believe that a lot of that is simply to check the box on the AUC and I have some issues with that.”* (Site # 11).

He also shared sites may not have the capacity to fully comply with the documentation due to the lack of staff and standardization of the documentation process.

Other low performing sites indicated that chart abstractors/analysts obtained the information, that the process could be streamlined, and that conflicting information obtained by the data abstractor reviews pre and post procedure was a problem. Documentation was also difficult when data could not be obtained due to separate medical records.

Finally, a couple of low performing sites believed it was the obligation of the referring physician to work the patient up appropriately before sending them for the procedure.

### Pressures (external/internal) and education

All sites had some form of external pressure, either from referring physicians, patients, insurance companies, or internal pressure from the quality assurance and safety standards of the organization. A medium performing site indicated that *“some physicians have lower threshold of when to send patient to cath lab”*. (Site 6) A high performing site used retrospective evaluations to identify trends in the inappropriate use of elective PCI.

The external pressure from insurance companies drove appropriateness by encouraging a high performing site to use AUC. The documentation was shared with the insurance company, forgoing the need for pre-authorization. On the other hand, patient pressure to have the procedure was identified as a barrier at both high and low performing sites when assessing appropriateness of an elective PCI. A high performing site shared they understood this pressure as patient satisfaction was very important. Finally, contending with referring physician pressure to do procedures was a difficult situation. A high performing site indicated it was difficult to get outside referring physicians up to speed with measures of appropriateness for an elective PCI. Another high performing site shared this was difficult because the referring physician was someone that was known and was an important source for referrals.

Education was used to improve appropriateness of PCIs within multiple organizations. Educating patients who believed they “needed” a PCI was identified as an important factor to facilitate AUC. A site used the SCAI website routinely when encountering resistance from patients. AUC education of the community physicians was identified as a requirement for both high and low sites. Educating the internal staff for appropriate documentation of the AUC was identified as an important step to ensuring successful implementation of AUC in their facility.

### Peer review process

High performing sites more often described having developed their own peer review processes to ensure appropriateness, team communication, and proper documentation. None of the medium performing sites described any type of peer-review processes.

One of the high performing sites created peer review sessions that included the full cath lab team and were based on AUC guidelines. These cardiac cath conferences occurred every 2 weeks. They also had mortality reviews every 4 months that were open to everyone and a quality program that was organized by the cath lab director. Multiple sites had peer review and quality improvement meetings and processes that included their administration to monitor procedures that would encourage providers to do the “*right thing*” and continually improve processes. Another high performing site reported an informal process of regular communication within a small team of cardiologists.

Most of the low performing sites did not mention peer review processes. A few shared they had improvement processes such as *“workgroups looking at processes for quality and want to improve”* or their administration was involved with overall quality, referrals, and growth and development strategies. A low performing site indicated that their quality was driven by comparison to COAP benchmark reports.

## Discussion

Through the qualitative analysis of interviews with cardiologists, managers of cath labs, and directors of cardiology programs at 13 sites participating in the Washington State COAP, we identified factors that facilitated or impeded the implementation of the AUC to minimize inappropriate use of elective PCI. The overarching themes that emerged from this analysis included the importance of relationships and competition, the referral source for PCI, the locus of responsibility for the pre-procedural evaluation, documentation, education, and peer-review in the minimization of inappropriate PCI. These themes provide a framework to approach implementation of the AUC in support of high-quality patient selection.

To our knowledge, no prior studies have explored the perspectives of the clinical and operational personnel involved in the implementation of AUC for coronary revascularization. Studies were found of appropriate use criteria in other cardiovascular clinical areas such as coronary computed tomography angiography, myocardial perfusion imaging, and transthoracic echocardiography where researchers found using AUC to improve care delivery, education, and cost [16, 17]. Although specific tools have been suggested to support adherence to AUC, prior to our study, aspects of implementation have not been described [18].

We found most of the sites, high, medium, and low, believed the AUC were a positive step in improving patient care, beneficial to providers as a guidance tool and providing peer review processes. Our study contributes to the literature with strategies that may lead to improvements in patient selection and appropriate use of PCI, such as proper documentation, which was often supported with checklists and calculators.

Across all sites, education was identified to reduce the inappropriate use of PCI. This included educating patients, community physicians, interventional cardiologists, and staff as to exactly what the AUC are and why they are important. These educational components supported open discussions with outside referring physicians and patients to “*get them up to speed*” regarding the appropriateness of elective PCIs, obtaining the needed pre-procedural information from the providers, and encouraging appropriate documentation of the AUC by staff and cardiologists. The literature has identified education to be a key component in the adherence to AUC as well as educational interventions that could be generalized to other practice environments [19–21].

Education was also needed to help explain how the AUC for coronary revascularization are not intended to diminish clinical decision making [22–25]. In our study, multiple interventional cardiologists stated there needs to be more room for individual clinical judgement in the AUC. Yet, as with all clinical practice guidelines (CPG), some providers do not trust the AUC as they have concerns about how the criteria were derived [21, 22].

Prior studies have identified many factors that often impede implementation of CPG. Cabana, et al. [26] identified factors that may contribute to poor adherence of practice guidelines, including lack of: awareness, familiarity, agreement, self-efficacy, outcome expectancy, and the inertia of previous practice and external barriers. In a recent scoping review by Fisher et al., [27] similar factors were found, however organized into personal factors (physicians’ knowledge and attitudes), guideline-related factors (lack of evidence, complexity, lack of applicability, etc.), and external factors. As the AUC reflect a type of clinical practice guideline, we were attuned to evaluating these potential factors in the present study. Interestingly, our study found that all the participants we spoke with were very aware and familiar with the AUC, the clinicians were comfortable in implementing the criteria, and most believed it was beneficial. A few felt their clinical ability to determine appropriateness was being undermined. The bulk of the barriers to implementation of AUC were within organizational realm, rather than at the level of the provider.

The iterative nature of clinical guidelines has brought about with the revisions of the AUC guidelines using terminology to reflect real-world treatment patterns and replacing “inappropriate” with the term “rarely appropriate” [28]. We have used the prior terminology to be consistent with criteria applied at the time of study.

With increasing emphasis on value-based healthcare, there is interest in reducing the use of unnecessary care that contributes to healthcare cost without improving patient outcomes. Accordingly, AUCs have been employed as justification for reimbursement [24]. Within our study, we found external pressure from an insurance company contributed to implementation of the AUC at a high-performing site. At this site, AUC documentation was shared with the insurance company in place of pre-authorization.

Other factors emerged to influence the uptake of the AUC. One was that of competition for patients. In most high performing sites the majority of the patients were referred from cardiologists inside their organization. Low performing sites referrals originated from providers outside their organization and there was concern about offending referring cardiologists. Additionally, multiple sites identified patient pressure to have the procedure as a barrier and if told they were not appropriate candidates, patients may have chosen to go elsewhere to have the procedure and subsequent care.

Finally, the burden of documentation and staffing needs to comply with AUC measurement was identified at a low performing site. As an unintended consequence of AUC, the requirement of the documentation is one of the deciding factors for a site’s performance. While the AUC does not necessarily improve patient appropriateness but verifies it through documentation, the sites may appear as low performance sites if providers struggle to document AUC due to lack of time and supporting staff. Providers were aware of the boxes they needed to check but were struggling with the documentation process due to clinical priorities. A 2012 Kaiser Family Foundation study found that U.S. physicians spend more than 868 million hours annually on prior authorization activities, thus resulting in rising financial burden associated within obtaining and documenting AUC processes [16]. Therefore, potential solutions may include streamlining the process of clinical documentation to support AUC assessment concurrent to documentation of clinical care. Another solution may support more interoperability of EMRs across sites to facilitate documentation from inside and outside referrals. In addition, hiring more supporting staff to assist with AUC documentation process may eliminate administrative burden for providers and improve the overall AUC uptake.

Approaches to address factors identified in this study could be adopted by less successful sites to improve their selection of patients for elective PCI. First, organizations can support the process with implementation of collaborative environments that allow all staff to participate with questions and opinions. Other factors include 1) collaborative environments with referring providers to exchange educational information; 2) better staffing and training/education of the multidisciplinary cath lab teams regarding AUC; 3) direct interventional cardiologist involvement with the pre-procedural review process; 4) checklist-based algorithms for pre-procedural documentation; 5) utilization of sophisticated information/data collection systems; and 6) systems redesign to include insurance companies and payment models that reward value over volume [29]. Physicians can facilitate shared decision making with expanded information from the AUC that informs patients [29, 30].

Strengths of our study include the identification and assessment of opinions and perspectives of high, medium, and low performing PCI sites, in terms of appropriate patient selection for elective PCI, in the state of Washington. Our findings should be considered in light of the following potential limitations: First, there is the potential for social desirability bias to influence our results; i.e., colleagues responding in a certain way to please the interviewer. The interviewers were very explicit to identify themselves as professional researchers, not clinicians. We believe this minimized response bias. Also, sites with low volumes of elective procedures were not included in the sample; this and other factors resulted in 16 sites that were eligible for interviews. At four of the 13 sites that agreed to participate, we were not able to speak with a cardiologist, although we strongly pursued a cardiologist at each site. Three of the four sites where we were unable to speak with a cardiologist were low performing sites. These sites had access to their COAP results, which may have played a role in the decision of the cardiologist not to speak with us. Finally, further research is needed to understand the relationships between elective PCI volume and staffing levels.

## Conclusions

In conclusion, from interviews with cardiologists, staff, and administrators at hospitals performing PCI in Washington State, we identified specific strategies that facilitated the successful implementation of AUC. Understanding these factors provides a foundation for future efforts to improve patient selection and procedural appropriateness for elective PCI.

## Abbreviations

AUC: Appropriate Use Criteria
cath lab: Cardiac catheterization laboratories
COAP: Clinical Outcomes Assessment Program
CPG: Clinical practice guidelines
EMR: Electronic medical records
PCI: Percutaneous Coronary Intervention
SCAI: Society for Cardiovascular Angiography and Interventions
